# Diurnal Intraocular Pressure Fluctuation in Eyes with Angle-closure

**DOI:** 10.5005/jp-journals-10008-1178

**Published:** 2015-01-15

**Authors:** Shibal Bhartiya, Parul Ichhpujani

**Affiliations:** Senior Consultant, Department of Ophthalmology, Glaucoma Facility, Fortis Memorial Research Institute, Gurgaon, Haryana, India; Assistant Professor, Department of Ophthalmology, Glaucoma Facility, Government Medical College and Hospital, Chandigarh, India

**Keywords:** Intraocular pressure, Angle-closure glaucoma, Diurnal variation.

## Abstract

**Purpose:** To investigate diurnal intraocular pressure (IOP) fluctuation in eyes with angle-closure.

**Materials and methods:** Seventy-seven eyes of 77 newly diagnosed patients with angle closure [33 subjects with primary angle-closure suspects (PACS), 23 subjects with primary angle-closure (PAC), and 21 subjects with primary angle-closure glau-coma (PACG)] were enrolled after laser peripheral iridotomy for this prospective, cross-sectional study.

Goldmann applanation tonometry (GAT) was performed at 08:00, 12:00, 16:00, 20:00, and 04:00 hours. Mean diurnal IOP, peak IOP, trough IOP, and IOP fluctuation (peak-trough) were compared between groups.

**Results:** The mean age of the enrolled subjects was 56.8 ± 5.4 years, with 30 males and 47 females. Intraocular pressure fluctuation was significantly higher in the PACG (7.4 ± 2.8 mm Hg) and PAC (5.5 ± 2.3 mm Hg) groups compared with PACS subjects (4.4 ± 1.5 mm Hg). The highest IOP was recorded at 04:00 hours in all but two patients. Two PACS, 10 PAC and 8 PACG patients, with normal office hour IOP had IOP peaks > 21 mm Hg at night. Twenty-five percent patients (20/77) had abnormal IOP despite good office hour readings.

**Conclusion:** Primary angle-closure glaucoma and PAC eyes showed diurnal IOP fluctuations greater than 5 mm Hg in most subjects, with peak IOP recorded at 04:00 hours. Peak IOP was higher than office hour IOP recordings in subjects with angle-closure. A diurnal variation curve is recommended in these subjects, especially in cases with controlled IOP during office hours.

**How to cite this article:** Bhartiya S, Ichhpujani P. Diurnal Intraocular Pressure Fluctuation in Eyes with Angle-closure. J Curr Glaucoma Pract 2015;9(1):20-23.

## INTRODUCTION

Intraocular pressure (IOP) is the most important, and the only modifiable, known risk factor for glaucoma^1-8^ and consequently, most therapeutic interventions are directed at its modification.

## MATERIALS AND METHODS

In this prospective cross-sectional study, subjects older than 40 years were recruited from outpatient clinics of two tertiary care centers in North India, after attaining a written, informed consent.

The study adhered to the tenets of declaration of Helsinki and had the approval of the institutional review board. Angle-closure patients were classified according to the International Society for Geographical and Epidemiological Ophthalmology (ISGEO) definition of the disease.^9^

Three subgroups of angle closure subjects were recruited; the definitions of each subgroup in the study were as follows:

Primary angle-closure suspects (PACSs) were defined as those with eyes in which the posterior trabecular meshwork was not visible for at least 180° on non-indentation gonioscopy, with IOP of 21 mm Hg or less, healthy optic nerves, and normal visual fields.

Primary angle-closure (PAC) eyes were those that, in addition to nonvisibility of posterior trabecular mesh-work, had peripheral anterior synechiae (PAS), raised IOP (defined as an IOP of more than 21 mm Hg), or both, but without glaucomatous optic neuropathy.

Peripheral anterior synechiae was defined as abnormal adhesions of the iris to the angle that were at least half a clock hour in width, were present to the level of the anterior trabecular meshwork or higher, and were deemed to be present if apposition between the peripheral iris and angle structures could not be broken despite indentation gonioscopy. The extent of PAS was noted in clock hours.

Primary angle-closure glaucoma (PACG) eyes were those with PAC and glaucomatous optic neuropathy (defined as vertical cup-to-disk ratio 0.7 or more, cup-to-disk asymmetry of more than 0.2, focal notching, or a combination thereof) with compatible visual field loss.

All the enrolled subjects had undergone a laser peripheral iridotomy at least 2 to 3 weeks prior to the study visit and were not taking any medications. Topical steroids were used for a period of 7 days post laser peripheral iridotomy, and it was ensured that no residual inflammation was noted before phasing measurements.

Subjects with previous acute angle closure, secondary angle closure (uveitis, neovascularization, ocular surgery or trauma), prior intraocular or penetrating eye injury and those using contact lenses for refractive correction were excluded.

Those taking glaucoma medications or systemic medications that could possibly influence IOP, such as beta-blockers, steroids, and diuretics were also excluded.

Intraocular pressure was recorded at 0800, 1200, 1600, 2000 and 0400 hours using a calibrated Goldmann applantation tonometer (GAT).

0400 hours reading was taken within 5 to 15 minutes of waking up.

Static and indentation gonioscopy were performed at baseline using a Zeiss 4 mirror lens (Carl Zeiss, Thorn-wood, NY). Under the lowest level of ambient illumination that permits a view of the angle and at high magnification (×16-×25), the drainage angle was graded according to modified Shaffer’s convention in each quadrant. Under this convention, the angle width was graded as 4 for wide open with the ciliary body being visible and as 0 to represent a state where no angle structures are visible in the primary position. Slight tilting was permitted in the presence of convex iris configurations. Peripheral anterior synechiae was noted in clock hours.

Statistical package for social sciences (SPSS) Version 16.0 was used for statistical analysis. Mean diurnal IOP, peak IOP, trough IOP, and IOP fluctuation (peak-trough) were compared between groups.

Kappa was done for agreement between the two observers.

## RESULTS

Out of 77 eyes of 77 patients with angle closure disease following laser peripheral iridotomy included in the observational study, 33 (42.9%) were diagnosed with PACS, 23 (29.9%) PAC and 21 (27.3%) with PACG. All of the patients were of Asian Indian ethnicity, and there was a preponderance of females (47/77, 61.0%).

The average age of the patients increased with the increase in disease severity (p-value: 0.8, but this difference was not statistically significant. The mean interval after laser peripheral iridotomy was 10.3 ± 18.2 weeks (5-82 weeks). Kappa for agreement between observers was found to be substantial (Cohen’s kappa―0.632).

**Graph 1:**
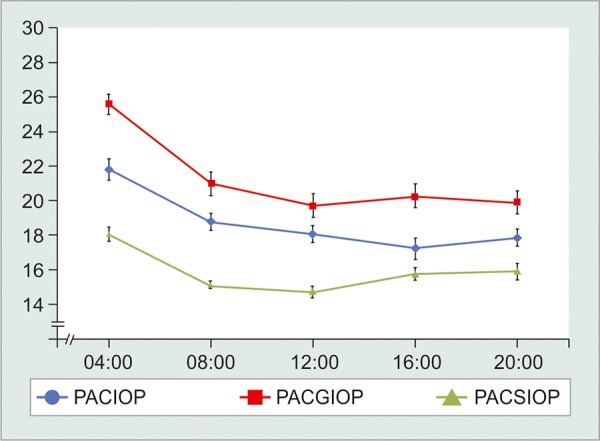
Mean IOP at various time points

Table 1 demonstrates the basic demographics and mean IOPs at each time point for each of the subgroups. Graph 1 illustrates the mean IOP (± SD) at each of the time points. The pattern of diurnal variation in IOP showed IOP peak in the morning hours at 4 AM in all three groups.

Table 2 illustrates the mean diurnal IOP, peak diurnal IOP, IOP fluctuation, and standard deviation of the IOP for study subjects. The mean fluctuation was found to significantly increase with increase in disease severity, being 4.39 ± 1.47 (2-9); 5.52 ± 2.29 (2-10) and 7.38 ± 2.83 (3-12) mm Hg for PACS, PAC and PACG, respectively.

Mean diurnal IOP (p = 0.000), peak diurnal IOP (p = 0.000) and IOP fluctuation (p = 0.001) showed significant differences between the three groups (Kruskal-Wallis test).

The correlation between peak IOP and fluctuation was found to be strong (Pearson’s correlation coefficient―0.658, p = 0.000).

Only three patients with PACG had fluctuations < 5; while 16 patients (16/21, 76.2%) had fluctuations ≥ 6 mm Hg. Only five patients (5/33, 15.2%) with PACS had fluctuations ≥ 6; while 22 patients (22/33, 76.2%) had fluctuations ≤ 5 mm Hg. The fluctuation in the PAC group was more evenly distributed, with 12 patients (12/23, 52.2%) having IOP fluctuation ≤ 5 mm Hg; and 11 patients (11/23, 47.8%) had fluctuations ≥ 6 mm Hg. Seventy percent (31/44) of eyes with PACG and PAC eyes showed diurnal IOP fluctuations greater than 5 mm Hg.

**Table 1:** **Table 1:** Baseline demographic data

		*PACS* *(n = 33)*		*PAC* *(n = 23)*		*PACG* *(n = 21)*	
Age ± SD		49.45 ± 8.07		56.60 ± 4.74		63.09 ± 7.25	
Gender (M:F)		12:21		9:14		9:12	
IOP at 0400 ± SD		18.09 ± 2.30		21.82 ± 2.97		25.61 ± 2.57	
IOP at 0800 ± SD		15.12 ± 1.38		18.78 ± 2.41		21.00 ± 3.06	
IOP at 1200 ± SD		14.72 ± 1.82		18.08 ± 2.27		19.71 ± 3.16	
IOP at 1600 ± SD		15.75 ± 1.96		17.21 ± 2.99		20.28 ± 3.30	
IOP at 2000 ± SD		15.90 ± 2.56		17.86 ± 2.36		19.90 ± 3.06	

**Table 2:** **Table 2:** Intraocular pressure profile in different forms of angle-closure

		*Mean* *IOP ± SD* *(Range)*		*Peak* *IOP ± SD* *(Range)*		*Fluctuation* *IOP ± SD* *(Range)*	
PACS		15.92 ± 1.61		18.18 ± 2.27		4.39 ± 1.47	
		(12.80-19.40)		(14-24)		(2-9)	
PAC		18.75 ± 2.17		21.91 ± 2.95		5.52 ± 2.29	
		(14.20-4.20)		(16-28)		(2-10)	
PACG		21.30 ± 2.45		25.71 ± 2.62		7.38 ± 2.83	
		(17.20-24.20)		(20-30)		(3-12)	
p-values		0.000		0.000		0.001	
(Kruskal-							
Wallis)							

**Table 3:** **Table 3:** Intraocular pressure peaks outside working hours with normal IOP during working hours

PACS		71.74%	
PAC		48.39%	
PACG		68.00%	

It is interesting to note that 73 IOP peaks at were noted at 4 AM and 2 peaks at 12 noon. In PACG patients, one peak was observed at 4 PM, as were 4 troughs.

Ten PAC (10/23, 43.48%), 8 PACG (8/21, 38.10%) and 2 PACS (2/33, 6.06%) patients had IOPs greater than 21 mm Hg after office hours, despite normal IOP between 8 AM and 4 PM. A total of 20 out of 77 patients (25.97%), therefore had abnormal IOPs despite good office hour IOP control; being 40% (18/44) of those with PAC/PACG.

Table 3 illustrates the number of patients of each subgroup who had IOP peaks (not necessarily > 21 mm Hg) after working hours, despite normal IOP during working hours.

## DISCUSSION

This study investigated the diurnal IOP fluctuations in eyes with various subtypes of angle-closure disease as classified by the ISGEO definition, using GAT, which is the current gold standard for tonometry.

The mean fluctuation in IOP was found to significantly increase with increase in disease severity, being 4.39 ± 1.47; 5.52 ± 2.29 and 7.38 ± 2.83 mm Hg for PACS, PAC and PACG, respectively. A significant difference between the mean diurnal IOP, and peak diurnal IOP was also noted between the 3 groups, with a strong correlation between peak IOP and fluctuation.

Seventy percent of PACS and PACG, and 50% of PAC patients had off office hours peaks (not necessarily > 21 mm Hg), emphasizing the need for diurnal variation curves in these patients.

As many as one-fourth of the patients had above normal IOPs despite good office hour IOP control; being 40% (18/44) of those with PAC/PACG. It is also of great clinical relevance that 73 IOP peaks at were noted at 4 AM.

In the only other study to investigate diurnal IOP fluctuations in eyes with various subtypes of angle-closure disease only office hour IOP using noncontact air-puff tonometry at hourly intervals between 8 AM and 5 PM. The authors also found that IOP fluctuation increases with severity of the angle-closure disease. Intraocular pressure fluctuation was significantly higher in PACG (5.4 ± 2.4 mm Hg) and PAC (4.5 ± 2.3 mm Hg) subjects compared with PACS subjects (3.7 ± 1.2 mm Hg) and normal controls (3.8 ± 1.1 mm Hg), with highest IOP found in the early morning.

The difference in the fluctuations reported by Baskaran et al maybe attributed to the fact that the study did not evaluate nocturnal IOP, and there is sufficient evidence that the peak circadian IOP is recorded between 2 AM and 4 AM in most patients.^10^

Barkana et al reported that peak 24-hour IOP was higher than the peak IOP noted during office hours in more than 60% patient.^11^

The combined PACG and PAC group had more than twice the risk of having IOP fluctuation of more than 3 mm Hg compared with the combined PACS and normal group. They also reported that the extent of PAS and visual field pattern standard deviation (PSD) were found to be associated with greater IOP fluctuation. The authors reported that PACG eyes seemed to show a pattern of afternoon IOP trough; however in our study, one peak was observed at 4 PM, as were 4 troughs.

Sihota et al on the other hand, had reported more IOP peaks in afternoon hours for PACG subjects.^12^ They also did not measure nocturnal IOP, with pressures recorded at 7 AM, 10 AM, 1 PM, 4 PM, 7 PM and 10 PM. They reported a diurnal fluctuation of > 8 mm Hg seen in 30%, >6 mm Hg in 85% eyes with PACG. The reported fluctuation was 4.83 ± 2.46 mm Hg in normal, 7.69 ± 3.03 mm Hg in PACG, and 8.31 ± 2.58 mm Hg in POAG group, with the difference between POAG and PACG not being significant statistically.

The limitation of our study is that the extent of PAS and degree of visual field loss as measured by PSD which are known to be associated with IOP fluctuation were not ascertained. Also, the use of GAT meant that this was not a habitual position DV curve, and no IOP measurements were performed between 2000 and 0400 hours. The study is also limited by its small sample size, and the fact that only angle closure subjects with previous LPI enrolled, which is not representative of the natural history of the disease. Also, the effect of LPI on IOP fluctuation is not known.

## CONCLUSION

- Primary angle-closure glaucoma and PAC eyes showed diurnal IOP fluctuations greater than 5 mm Hg in most subjects
- Peak IOP recorded at 0400 hours in most subjects
- Intraocular pressure fluctuation increases with severity of the angle-closure disease
- Peak IOP was higher than office hour IOP in 25% subjects overall; and 40% of those with PAC/PACG
- A diurnal variation curve may especially be useful in cases with continued progression despite controlled IOP during office hours.
